# Dynamic transcriptomic profiling of dorsal root ganglia reveals stage-specific mechanisms in diabetic neuropathic pain

**DOI:** 10.3389/fnmol.2026.1870499

**Published:** 2026-08-05

**Authors:** Qingping Zhang, Mingzhu Zhai, Jing Yang, Xiaoyan Qi, Pengxu Cang, Aiping Yuan, Shitao Zhang, Chen Liang, Zhou Qi, Shiwen Guo

**Affiliations:** 1 Department of Neurosurgery, The First Affiliated Hospital of Xi’an Jiaotong University, Xi’an, China; 2 Department of Neurosurgery, Affiliated Nanshan Hospital of Shenzhen University (Shenzhen Nanshan People’s Hospital), Shenzhen, China; 3 Center for Medical Experiments (CME), Shenzhen University of Advanced Technology General Hospital, Shenzhen, China; 4 Department of Endocrinology, Affiliated Nanshan Hospital of Shenzhen University (Shenzhen Nanshan People’s Hospital), Shenzhen, China

**Keywords:** diabetic peripheral neuropathy, dorsal root ganglion, KEGG, RNA-sequence, transcriptome

## Abstract

Diabetic peripheral neuropathy (DPN) is the main clinical challenge faced by patients in the middle and advanced stages of diabetes. Due to the unclear cellular and molecular mechanisms, despite decades of in-depth research, its treatment methods are still limited. This study delineates the dynamic transcriptomic landscape of dorsal root ganglia (DRG) in streptozotocin (STZ)-induced diabetic male rats, integrating behavioral phenotyping and cross-model comparisons. We observed phenotypic heterogeneity, with only 60% of STZ-treated rats developing diabetic neuropathic pain (DNP group). In comparison, 37% remained pain-free (non-DNP group) despite comparable hyperglycemia and weight loss. RNA sequencing revealed stage-specific molecular signatures: early DNP (4 weeks post-STZ) involved PI3K-Akt/Ras signaling pathways, whereas late DNP (8 weeks) implicated several virus infection and neuroactive ligand-receptor pathways. Strikingly, 72% of differentially expressed genes (DEGs) at 8 weeks were unique to chronic DNP maintenance. Cross-model analysis demonstrated little overlap between DNP, bone cancer pain, and nerve injury models (<1% shared DEGs), with only Serpina3n universally upregulated. Functional annotations highlighted dysregulated extracellular matrix remodeling, immune receptor activity, and virus-related pathways unique to DNP progression. These findings reveal (1) intrinsic phenotypic variability in diabetic neuropathy progression, (2) temporally distinct pathogenic mechanisms governing DNP initiation versus persistence, and (3) a unique transcriptional fingerprint distinguishing DNP from other neuropathic pain etiologies. This work provides a roadmap for stage-specific therapeutic targeting and underscores the necessity of precision approaches in diabetic neuropathy management.

## 1 Introduction

Diabetic peripheral neuropathy (DPN) affects approximately 30% of individuals with long-standing diabetes, manifesting as sensory loss, chronic pain, and an elevated risk of limb amputations (Elafros et al., 2022; GBD 2021 Forecasting Collaborators, 2024). Despite decades of research, the molecular mechanisms driving DPN remain incompletely resolved, and therapeutic interventions are largely palliative (Eid et al., 2023).

The pathological mechanism of DPN involves a combination of multiple factors such as metabolic disorders, microvascular lesions, mitochondrial damage, homeostasis of growth factors, and neuroinflammation (Abd Razak et al., 2024; Baum et al., 2021; Bowling et al., 2015; Calcutt, 2020; Eid et al., 2023; Liu C. et al., 2025). Traditional paradigms emphasize hyperglycemia-induced mitochondrial dysfunction, oxidative stress, and microvascular damage as primary drivers of axonal degeneration (Calcutt, 2020). However, growing evidence suggests a crucial role of immune cells in diabetic nerve repair and tissue healing. For instance, recent research shows that CCR2+ macrophages infiltrate the sciatic nerves of type II diabetic mice before axonal degeneration, which protects against sensory axon loss in peripheral neuropathy (Hakim et al., 2025). Immunometabolic stress-induced dysregulated activation of mast cells is a potential driver in the progression of DPN (Yao et al., 2025). Furthermore, neuro-immune interactions have shown the potential to treat impaired tissue that undergoes dysregulated neuro-immune interactions (Lu et al., 2024). Nociceptive sensory neurons have exerted both protective and harmful effects as immunoregulators and depending on the immune microenvironment (Baral et al., 2018; Hanc et al., 2023; Yang et al., 2022). These findings highlight that the dual roles of immune cells and neuro-immune crosstalk in diabetic neuropathy, both protective and detrimental, are critically shaped by microenvironmental dynamics, offering a novel hint to restore immune homeostasis for DPN.

High-throughput RNA sequencing (RNA-seq) has revolutionized our understanding of neurological-disease-driven chronic pain by enabling unbiased profiling of gene expression networks (Sun et al., 2017; Zhai et al., 2021, 2024). Regarding diabetic neuropathy, transcriptomic studies have predominantly targeted sural nerves (Ferreira et al., 2024) or spinal cord tissues (Du et al., 2019), revealing dysregulation of pathways related to metabolic processes, calcium ion transport, and retrograde endocannabinoid signaling. Recent studies have shown that MAAC neurons (marked by Fxyd7+/Atp1b1+) in DRG of painful DPN models, which crosstalk with satellite glial cells to mediate neuroinflammation and abnormal axonal growth (Zhou et al., 2022). A single-cell transcriptomic analysis identified distinct cell subpopulations associated with diabetic neuropathy in the DRG (Guo et al., 2024). Emerging evidence suggests that nonneuronal skin cells may modulate nociceptor function in a high-fat diet mouse model (Pacifico et al., 2026). Together, these results suggest that altered neuron-immune cell communication may contribute to DPN.

DRG neurons are the primary sensory neurons in the somatosensory afferent pathway. They convert and integrate various stimuli into electrical signals and transmit harmful information from the peripheral nervous system to the central nervous system, acting as the primary transfer stations for abnormal pain signals during the occurrence and development of DPN. Emerging RNA-seq evidence indicates that immune molecules and responses from macrophages and immune cells in DRG contribute to the induction and maintenance of DPN (Eid et al., 2024; Hall et al., 2022; Hinder et al., 2018). Therefore, exploring the changes at the overall transcriptome level of genes in DRG and analyzing the changes in the overall mRNA levels of each signaling pathway are helpful for identifying important signaling pathways and potential therapeutic targets involved in the occurrence and development of DPN.

Previous studies have reported bulk or single cell transcriptome alteration and in the lumbar DRGs of DPN rat model (Athie et al., 2018; Guo et al., 2024). However, none of these studies distinguished and comparison the transcriptome differences between painful and non-painful phenotype in DPN model, and the dynamic transcriptome changes as the disease progressed were still unclear. In the current study, multi-staged transcriptome profiling at different time points (4 and 8 weeks for the DNP group, and 8 weeks for non-DNP and vehicle control groups) in DRG was depicted by conducting RNA-Seq to capture the dynamic changes in gene expression during the initiation and development of DNP. Our results revealed that different sets of differentially expressed genes (DEGs) are involved in the initiation and maintenance of DNP. In the early stages, the PI3K-Akt and Ras signaling pathways were significantly enriched, indicating their role in the induction of DNP. In the later stages, pathways related to virus infection, such as Herpes simplex virus 1 and Epstein-Barr virus, became prominent, suggesting a shift in the molecular processes driving DNP progression. By comparing the transcriptome profile of DNP with that of nerve injury and bone cancer pain models, we found high uniqueness in the DEGs among these models. These results present a global and comprehensive perspective of transcription profile alterations in DRG and provide a new insight into the underlying mechanisms of DNP and guidance for rational clinical medication.

## 2 Materials and methods

### 2.1 Ethics statement and animals

All procedures were performed following the regulations of the ethics committee of the International Association for the Study of Pain and the Guide for the Care and Use of Laboratory Animals. The experiments were designed to minimize suffering and the number of animals used and approved by the Ethics Committee of Shenzhen Nanshan People’s Hospital (No.: NSHP-JY-25020). The study utilized Sprague-Dawley rats obtained from the Guangdong Medical Laboratory Animal Center. Male Sprague-Dawley rats with an initial body weight of 160–180 g, 6–8 weeks old upon arrival, were housed in pairs per cage under controlled environmental conditions, with a room temperature maintained at 20–24 °C and a 12-h light/dark cycle. Animals had free access to food and water throughout the experimental period. The experimental animals were anesthetized with isoflurane (5% induction, 2% maintenance, RWD Life Science, China) during dissection procedures, and euthanized by pure CO2 asphyxiation with a flow rate set 20 L/min. Briefly, the experimental animals were placed in independent ventilation cages, which were then placed in fume hoods. Carbon dioxide was released through gas pipes to suffocate the experimental animals to death.

### 2.2 Model of DPN

Type 1 diabetes-associated DPN was induced via intraperitoneal injection of streptozotocin (STZ, 572201, Sigma Aldrich, USA). In brief, male Sprague-Dawley rats (160–180 g) were randomly divided and fasted for 12 h prior to treatment. STZ was dissolved in 50 mM sodium citrate buffer (pH 4.5) and administered within 15 min of preparation. A single intraperitoneal dose of 70 mg/kg STZ was injected to establish the diabetic models, and the vehicle group received an equivalent volume of sodium citrate buffer (Ye et al., 2021). After 7 days of intraperitoneal injection of STZ solution, the blood glucose from the tail vein body weight and body weight were monitored during the experiment. Rats with blood glucose concentrations ≥16.7 mM were classified as diabetic models, while those that failed to meet this criterion were excluded (Ye et al., 2021). Personnel conducting behavioral assays and preparing DRG tissues for RNA-sequencing were blinded to the surgical procedures. A total of 20 animals received STZ treatment, and 8 for the vehicle group.

### 2.3 Assessment of DPN-related pain behaviors

Mechanical allodynia and thermal hyperalgesia were assessed in STZ- and vehicle-treated rats using mechanical and thermal stimulation paradigms. Prior to behavioral testing, animals were maintained on a standard diet for 1 week, then followed by a 12-h fasting period. All tests were conducted under double-blind conditions between 9:00 and 21:00 h to ensure experimental rigor. Rats were classified as belonging to the DNP group if they exhibited significant and sustained reductions in both mechanical withdrawal threshold (≥35% decrease from baseline) and thermal withdrawal latency (≥20% decrease from baseline) for at least two consecutive weeks starting from week 2 post-STZ injection. This criterion was used to increase the consistency of behavioral phenotypes and transcriptome differences by referring to the common practice in neuropathic pain models (Smith et al., 2018). Rats with hyperglycemia (blood glucose ≥16.7 mM) but without meeting these pain behavior criteria by week 8 were classified as the non-DNP group.

#### 2.3.1 The measurement of mechanical allodynia

Mechanical allodynia was evaluated by measuring the decrease in mechanical withdrawal threshold on the plantar surface of the hind paws. The paw withdrawal response was assessed using von Frey monofilaments (Aesthesio, Ugo Basile, Italy) following protocols described in prior studies (Chaplan et al., 1994; Zhang et al., 2013). Briefly, rats were first transferred to the behavioral testing room and allowed to acclimate for 30 min. Each animal was then positioned individually under a ventilated Plexiglas chamber (20 × 15 × 10 cm^3^) with a 5 × 5 mm^2^ metal mesh floor for 10 min. Von Frey filaments were applied perpendicularly to the central plantar surface of each hind paw, exerting uniform pressure until the filament curved 45 degrees and held for around 2 s. A rapid hind limb retraction was defined as a positive mechanical allodynia response. To minimize stimulus-induced bias, a 30-s interval was maintained between consecutive tests. The Chaplan up-down method was used in this measurement, and the results were quantified and presented as the 50% paw withdrawal threshold. Data recording and analysis followed established methodologies referring to previous reports (Chaplan et al., 1994).

#### 2.3.2 The measurement of thermal hyperalgesia

Thermal hyperalgesia was evaluated by the significant decrease in the latency of hind paw withdrawal to radiant heat stimulus, which was measured by an analgesia meter (Model 390G; IITC Life Science) following a protocol adapted from previous studies (Zhang et al., 2013). In brief, rats were first acclimated to the behavioral testing room for 30 min. Each animal was then placed individually in a ventilated Plexiglas chamber (20 × 15 × 10 cm^3^) on a temperature-controlled glass surface maintained at 37 °C for 10 min. At each test, a focused radiant heat source was applied to the mid-plantar region of each hind paw. The stimulus automatically terminated upon paw withdrawal, with the latency recorded electronically (capped at 25 s to prevent tissue injury). Each paw was tested four times at 5–8 min intervals, and the average latency was used for statistical analysis. The glass surface temperature and stimulus intensity were standardized across all experimental sessions.

### 2.4 Total RNA extraction

After cervical dislocation, L4-L5 DRGs were dissected from rats in the STZ or vehicle treated groups, with all DRG tissues from one animal pooled as a single sample. DRG samples were collected at 4 and 8 weeks post-STZ treatment, and 8 weeks post-vehicle administration. For RNA-sequencing analysis, DRG tissues were collected from 4 animals per group at each time point. Specifically, the sample sizes for each group were as follows: Vehicle (8 weeks, *n* = 4), Non-DNP (8 weeks, *n* = 4), DNP 4w (4 weeks, *n* = 4), DNP 8w (8 weeks, *n* = 4). All collected samples passed quality control and were included in the final analysis. Total RNA was isolated using Trizol reagent (Sigma-Aldrich, USA) according to the manufacturer’s protocol, followed by chloroform extraction and isopropanol precipitation. The extracted RNA was stored at −80 °C until further use.

### 2.5 RNA sequence and data analysis

The preparation of the cDNA libraries and the transcriptome sequencing for all samples were completed by the Beijing Genomics Institute (BGI, Shenzhen, China). The cDNA derived from RNA fragments was paired and sequenced using the high-throughput sequencing platform BGISEQ-500. On average, approximately 33 M raw reads were obtained for each sample. Raw reads were quality-filtered by SOAPnuke to remove adapters, low-quality reads and reads with excessive ambiguous N bases. Then, the clean reads were aligned to the reference database using the HISAT/Bowtie2 tool (Zhang et al., 2013). The gene expression was quantified using the RSEM software package (Li and Dewey, 2011). Based on the gene expression levels, the DEGseq2 algorithm was used to identify differentially expressed genes (DEGs) between groups. Gene ontology (GO) analysis and Kyoto Encyclopedia of Genes and Genomes [KEGG (Kanehisa and Goto, 2000; Kanehisa et al., 2025; Kanehisa, 2019)]-based gene functional annotation clustering analysis were performed by the ClusterProfiler (Yu et al., 2012) with a modified Fisher’s exact test followed by Benjamini–Hochberg multiple hypothesis testing correction using Rattus norvegicus as background, default options, and annotation categories. The functional annotation analysis was performed using over-representation analysis. Only genes satisfying |log2FC| ≥ 1, q ≤ 0.001 were submitted to GO/KEGG enrichment. The original data has been submitted to the Sequence Read Archive (SRA) database with an accession number PRJNA1272274. Public transcriptomic data downloaded from GEO were processed with standardized, uniform workflows matching our in-house RNA-seq pipeline to guarantee cross-dataset comparability.

### 2.6 Statistical analysis

Behavioral data were represented as means ± SEM. Statistical analysis of behavioral tests were performed with two-way ANOVA followed by Sidak’s multiple comparisons test using Prism 8 software. Two-tailed *P* values less than 0.05 were considered to be significantly different. Statistical analyses of transcriptome data were conducted using the R language (version 4.3.2). Statistical tests used are indicated where the tests are performed. Correction for multiple testing is applied whenever multiple tests are involved, along with a statement indicating which method of correction is used. *P* values less than 0.05 were considered to be significantly different for each test unless specifically indicated at the location of the test.

## 3 Results

### 3.1 Painful behavioral manifestations and pathoglycemia following STZ treatment

**FIGURE 1:**
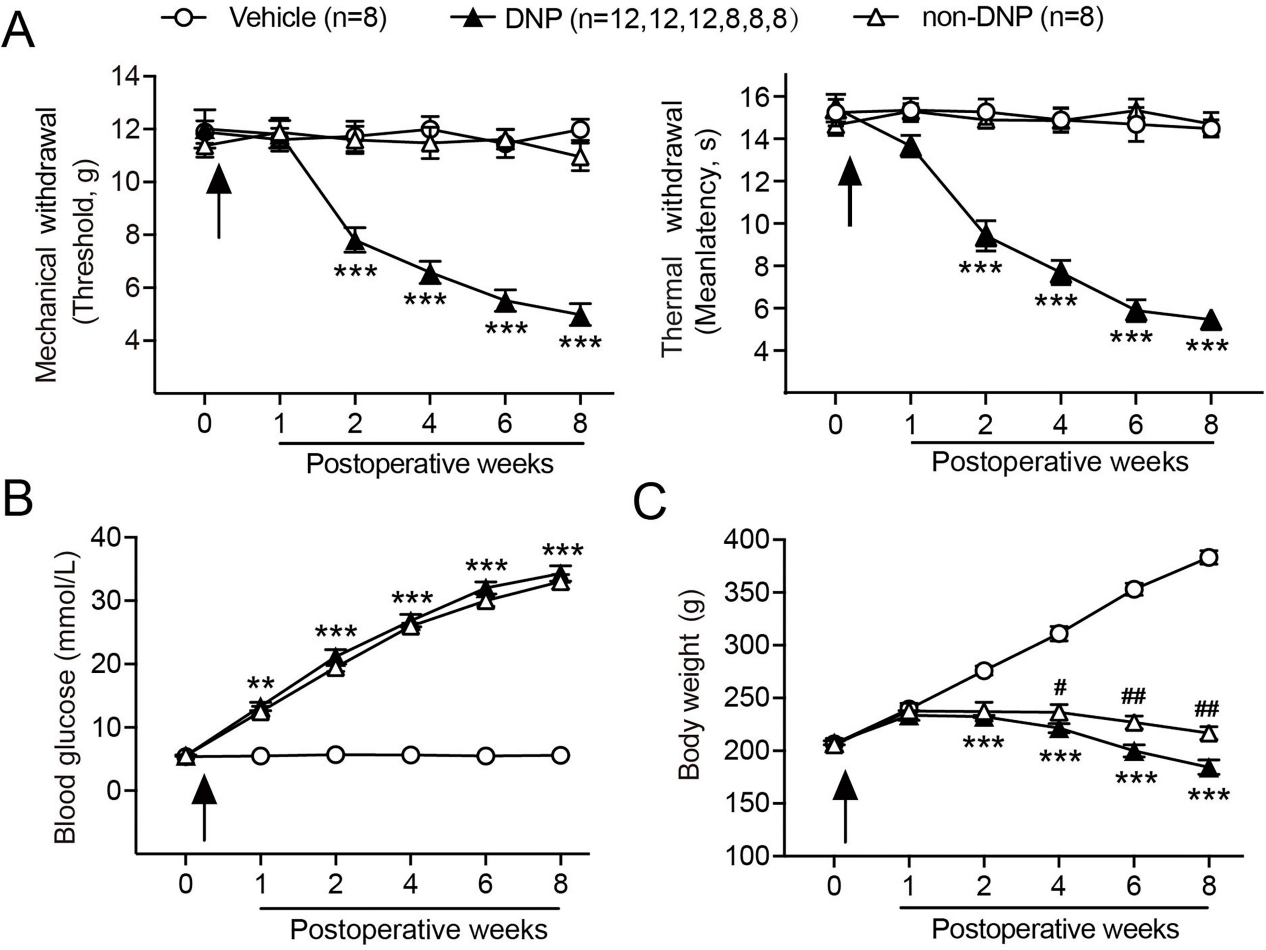
STZ-induced painful behaviors and changes in blood glucose and body weight in rats. **(A)** STZ-induced mechanical (Left panel) and thermal (Right panel) hypersensitivity manifested as lowered mechanical threshold shortened latency of thermal withdrawal. Two-way repeated measure ANOVA, ***P < 0.001 *vs.* vehicle group. **(B, C)** STZ-induced increased levels of blood glucose **(B)** and decreased body weight **(C)** in rats. Two-way repeated measure ANOVA, **P < 0.01, ***P < 0.001 vs. vehicle group. ^#^*P* < 0.05, ^##^*P* < 0.01 vs. DNP group. The arrows represent the STZ injection.

We examined the painful syndromes and alterations of blood glucose levels and body weight in rats with intraperitoneal injection of STZ. Of the 20 animals treated with STZ, 12 of the STZ-treated rats exhibited painful behaviors, as manifested by mechanical allodynia (Figure 1A, left panel) and thermal hyperalgesia (Figure 1A, right panel), starting 2 weeks after the injection, as shown in the DNP group. The other 8 rats showed increased blood glucose but no behavioral change after 8 weeks of STZ treatment, as shown in the non-DNP group (Figure 1A) compared to the vehicle group. We also traced and examined the blood glucose and body weight for 8 weeks (Figure 1B,C). The level of blood glucose (Figure 1B) was increased from 1 week after the STZ injection, and the body weight was decreased (Figure 1C) from 2 weeks after the STZ injection in both DNP and non-DNP rats. These results show clear behavioral signs, pathoglycemia, and weight loss in rats following STZ treatment.

### 3.2 An overview of DEGs profiles in DRG of DNP rats

**FIGURE 2:**
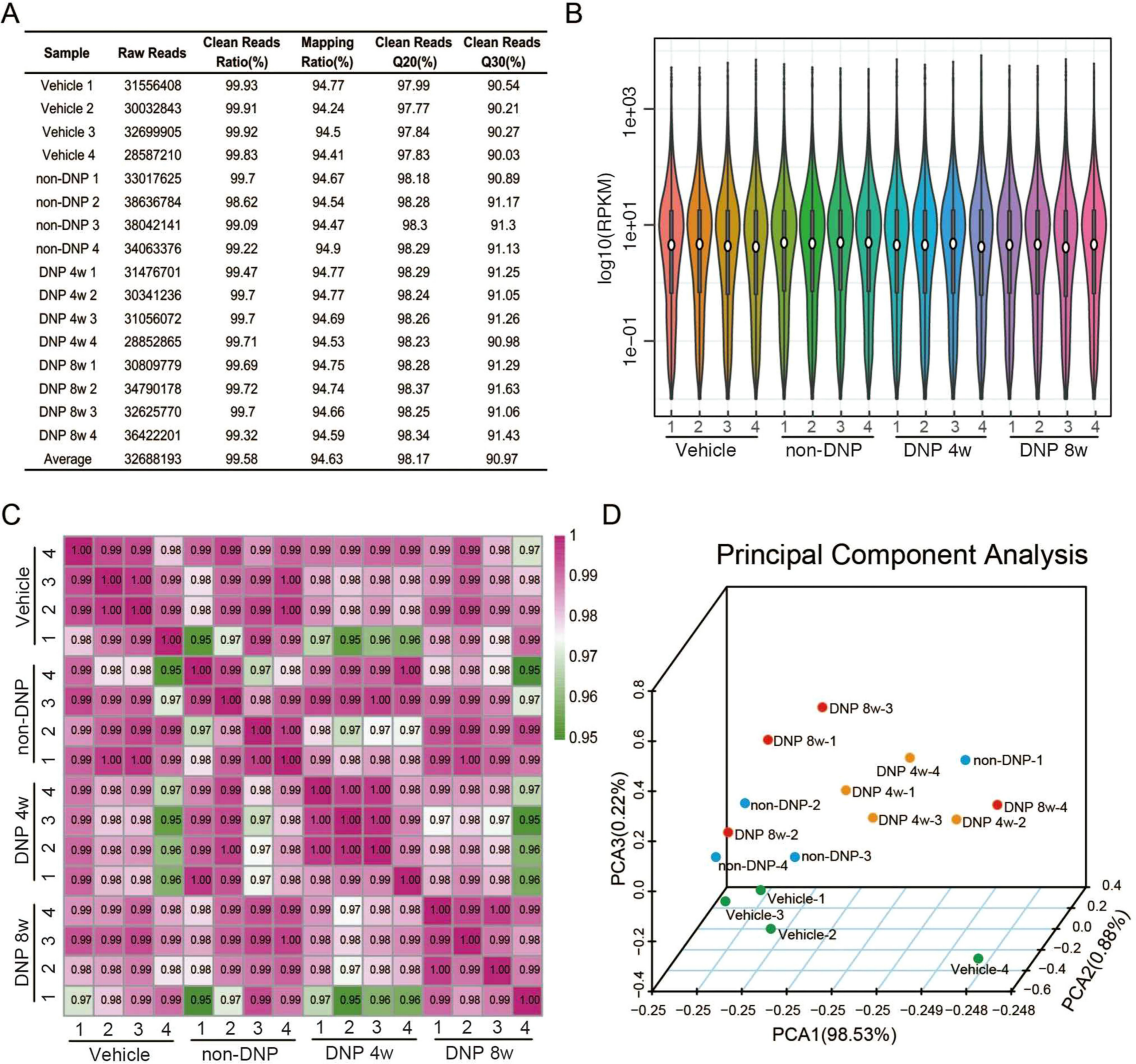
The quality of RNA-seq data from each of the DRG samples. **(A)** Summary of raw RNA sequencing data set from 16 samples, including raw reads number, clean reads ratio, mapping ratio, and percentage of clean reads, as well as Q20 (Phred quality scores Q) and Q30. **(B)** Distribution of gene expression levels in each of the samples. RPKM: Reads Per Kilobase Million. **(C)** Heatmap of the correlations between every two samples from each of the groups with the Pearson test. **(D)** Principal component analysis (PCA) of all the samples.

To evaluate the dynamic change of transcriptome profiles in DRG after STZ treatment, we performed RNA-Seq and gene analysis to detect gene expression patterns at different stages of DNP development. The transcriptome data were plotted at different time points, 4 and 8 weeks after STZ treatment for the DNP group, and 8 weeks after STZ treatment for the non-DNP and vehicle control groups. L4–L5 DRGs from bilateral of an animal in the different groups were collected as one sample for the subsequent transcriptome analysis. The summary of quality control of the raw RNA sequencing data set is shown in Figure 2A. On average, about 32,688,193 clean reads were collected and mapped to the reference genome using HISAT after filtering low-quality data and with a mapping ratio of around 94∼95%. The comparison of gene expression levels across sample groups, along with the uniformity in gene distribution dispersion for each sample, indicates that the samples are indeed comparable (Figure 2B). The correlation of gene expression levels among samples serves as a critical criterion for assessing the reliability of the experiments and the appropriateness of the selected samples. We calculated the correlation values between every pair of samples based on normalized expression results and generated a correlation heat map, and all pairs of samples had correlations greater than 0.9 (Figure 2C). Besides, the principal component analysis (PCA) was performed. DRG tissues collected from groups of Vehicle control and those 4 and 8 weeks after STZ treatment show intra-group aggregation (Figure 2D).

**FIGURE 3:**
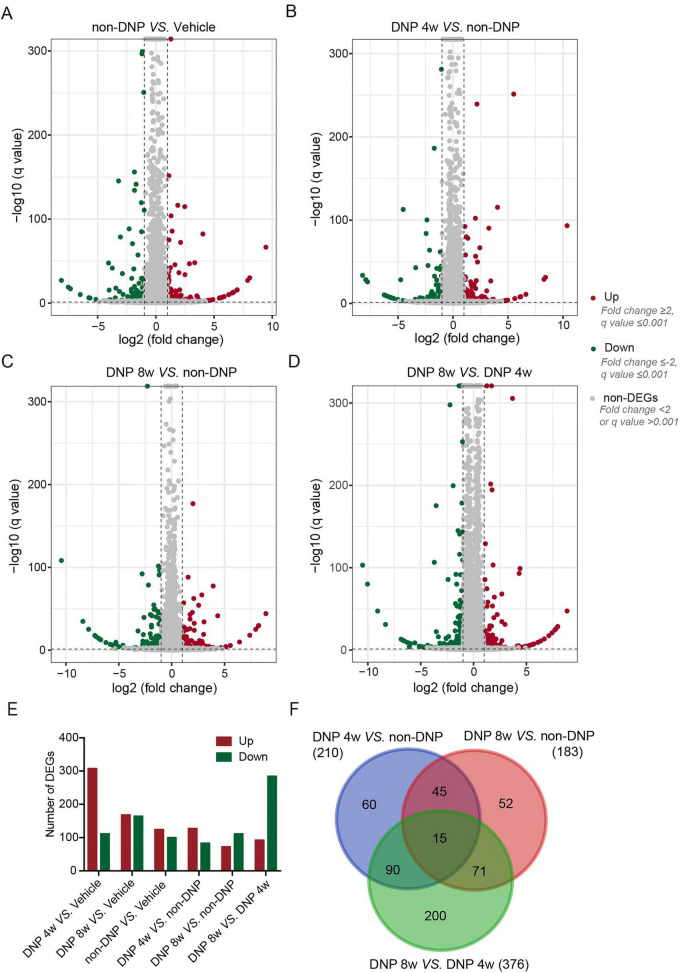
Dynamic alterations of DEGs in DRG after STZ treatment. **(A–D)** Volcano plot of all the differentially expressed genes from pairs of comparable groups. Log2 (fold change) is plotted as the abscissa and -log10 (Corrected *P* value) is plotted as the ordinate. Each dot represents a single gene (red dots = up-regulated genes; green dots = down-regulated genes; gray dots = genes with no significant difference). **(E)** Comparison of up- and down-regulated DEGs between groups. **(F)** A Venn diagram presents DEGs that are unique or shared in each of the paired groups.

Next, we examined transcriptome changes in the different stages of DNP development. DEGs screening was performed to filter the DEGs with the cut-offs of |log2 FC| ≥ 1 and normalized *P* value, q value ≤ 0.001 between two comparable groups for further functional analysis. The representative distributions of up- and down-regulated or no-change genes between the compared groups were shown in the volcanoes in Figures 3A–D, in which the red and green dots represent up- and down-regulated genes, respectively. In the non-pain group, when there was no painful behavior exhibited, there were 124 genes up-regulated and 100 genes down-regulated compared to the vehicle group (Figures 3A,E). On week 4 after STZ treatment when DNP had been well developed (also see Figure 1A), there were 127 genes up-regulated and 83 genes down-regulated compared to the non-DNP group (Figures 3B,E), and there were 307 (up-regulated) and 111 (down-regulated) DEGs compared to the vehicle group (Figure 3E and Supplementary Figure 1A). Furthermore, there were 72 genes up-regulated and 111 genes down-regulated detected on week 8 after STZ treatment compared to the non-DNP group (Figures 3C,E), and there were 168 (up-regulated) and 164 (down-regulated) DEGs compared to the vehicle group (Figure 3E and Supplementary Figure 1B). We also noted that there were 92 DEGs up-regulated and 284 DEGs down-regulated on week 8 of the DNP group compared to week 4 of the DNP group after STZ treatment (Figures 3D,E). These results indicate that DEGs in DRG a partially involved in the development of DNP. These gene expression alterations are synchronized with the development of DNP, suggesting the involvement of DEGs in different stages of DNP development. We also analyzed the unique or shared DEGs associated with DNP between each paired group, and the number of DEGs was represented by a Venn diagram (Figure 3F). Compared to the non-DNP group, in weeks 4 and 8 after STZ treatment, 150 (90+60) and 123 (71+52) genes were found unique, while 60 genes were shared between the two examined days. These results showed that 105 (90+15) DEGs were involved in the early development of DNP after STZ treatment, while the majority of the altered genes, approximately 72% [271 (200+71) out of 376], were responsible for maintaining the late persistence of DNP (Figure 3F). It is worth noting that 97 of these DEGs are also associated with hyperglycaemia (Supplementary Figure 1C). These findings reveal that the major gene expression and functional changes occur in week 8 after STZ treatment, suggesting that the DEGs expression occurred in the late-stage of DNP and may play a decisive role in the maintenance of DNP.

Furthermore, we conducted a detailed analysis of the DEGs associated with hyperglycaemia. Four-way Venn analysis of DEGs from four comparison groups (Supplementary Figure 1D) revealed distinct transcriptional signatures between DNP VS. Vehicle and DNP VS. non-DNP comparison in both 4 and 8 weeks, with far more unique DEGs identified in DNP VS. Vehicle groups (213 for 4w, 150 for 8w) than DNP VS. non-DNP counterparts (70 for 4w, 55 for 8w). Only 13 DEGs were shared across all four datasets, and limited overlapping genes existed between 4-week and 8-week DNP VS. Vehicle comparisons, demonstrating time-dependent transcriptomic remodeling during DNP progression and enabling isolation of hyperglycemia-specific gene expression shifts in DRG tissues.

### 3.3 Analysis of TOP20 KEGG pathways

**FIGURE 4:**
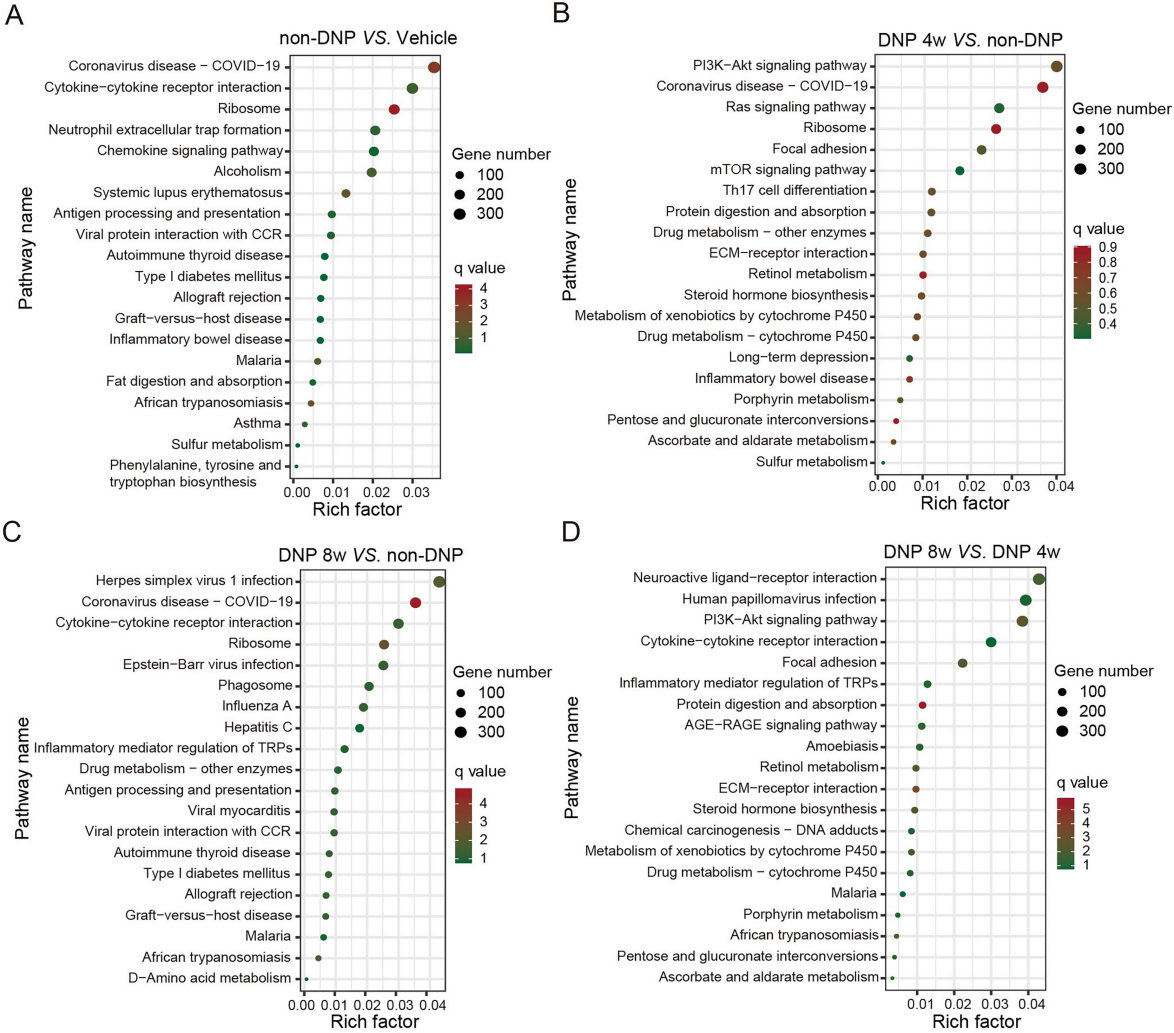
KEGG classifications of DEGs in DRG. **(A–D)** The comparison of pathway enrichment from pairs of comparable groups. The top 20 KEGG terms determined by the rich factor is plotted as the ordinate. The rich factor is plotted as the abscissa. The gene number involved in each pathway is represented by the size of the dots. CCR, cytokine and cytokine receptor.

To identify the major signaling pathways involved in the induction and development of DNP, we continued to analyze KEGG pathway enrichment of these DEGs. Our results showed that the significantly enriched DEGs were in the classifications of “Coronavirus disease-COVID-19”, “Cytokine-cytokine receptor interaction (CCRI)”, “Ribosome”, “Neutrophil extracellular trap formation” and “Chemokine signaling pathway” in non-DNP group compared to vehicle (Figure 4A), suggesting that these signaling pathways may be involved in non-painful diabetic peripheral neuropathy. In week 4 after STZ treatment, compared to the non-DNP group, the DEGs were significantly enriched in the “PI3K−Akt signaling pathway”, “Ras signaling pathway”, “Focal adhesion”, and “mTOR signaling pathway” (Figure 4B). Notably, the PI3K−Akt signaling pathway exhibited the most significant changes in week 4 after STZ treatment. However, In week 8 after STZ treatment compared to non-DNP group, the “Herpes simplex virus 1 infection”, “Epstein−Barr virus infection”, and “Phagosome” signaling pathways were significantly enriched, despite the other three signaling pathways enriched as same as the comparison of non-DNP and vehicle groups (Figure 4C). Finally, we compared the enrichment of DEGs between week 8 and week 4 after STZ treatment. The results showed that DEGs were significantly enriched in “Neuroactive ligand−receptor interaction”, “Human papillomavirus infection”, and “PI3K−Akt signaling pathway”. (Figure 4D). The unique and shared KEGG terms related to DNP (Supplementary Figure 2C) and hyperglycaemia (Supplementary Figure 2D) are shown in the Venn diagrams, respectively (Supplementary Figure 2). These results suggested that coronavirus disease, CCRI, and ribosome-related signaling pathways are the major processes in the responses to the STZ treatment, and PI3K−Akt and Ras signaling pathways play a major role in initiating the induction of DNP. In contrast, pathways related to virus infection, such as those involving Herpes simplex virus 1, Epstein−Barr virus, and human papillomavirus, predominantly contribute to the later stages of DNP progression. Notably, neuroligand-receptor activity, viral infection, and dysregulation of the PI3K-Akt signaling pathway, which is closely linked to neuronutrition and metabolism, constitute three crucial factors driving the development of DNP.

### 3.4 Gene ontology analysis of DEGs

**FIGURE 5:**
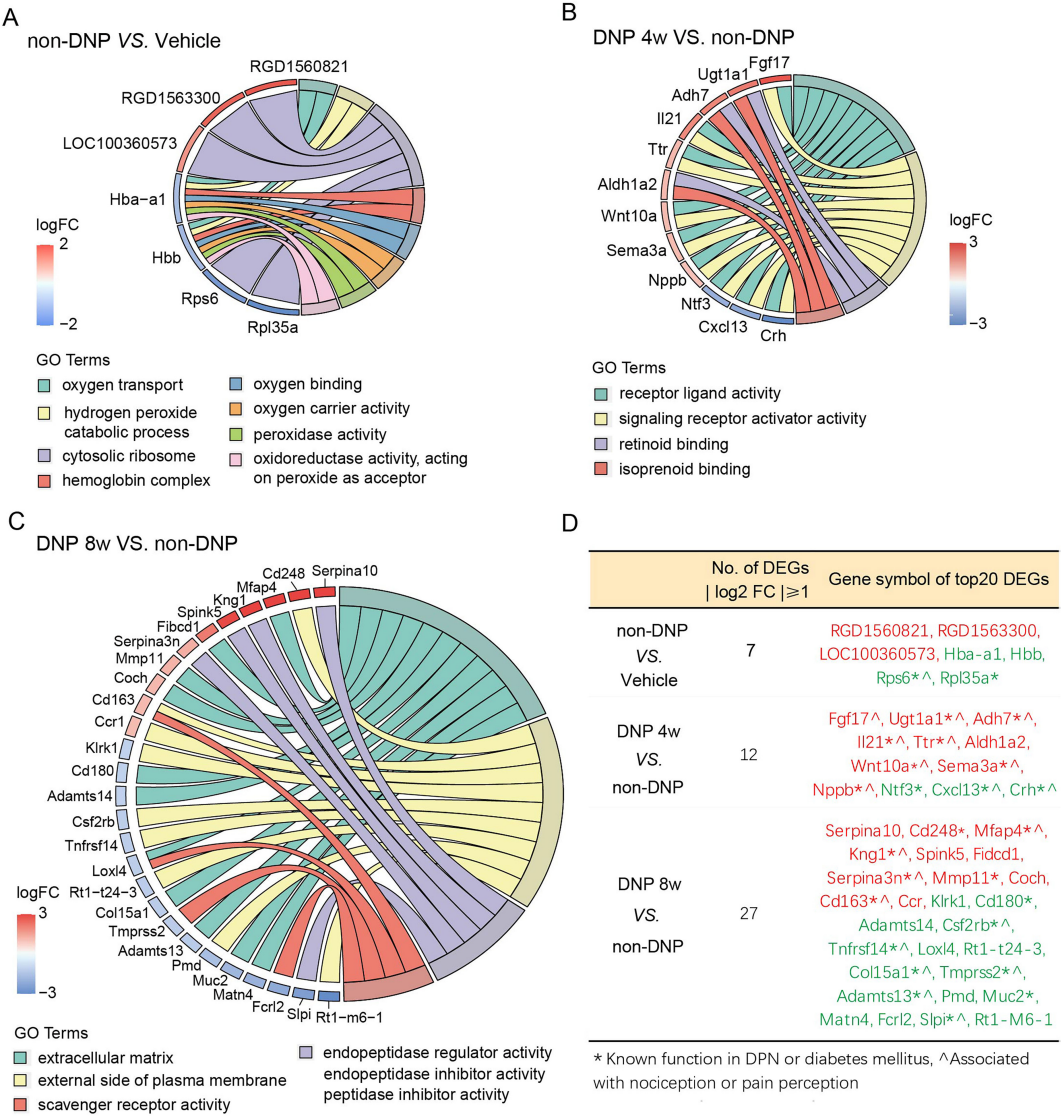
Functional analyses of DEGs in DRG from different stages. (**A–C)** Gene ontology (GO) analysis showing the enrichment of DEGs. **(D)** Functional analysis of the top 20 differential genes at different stages of DNP development. The red font highlights the upregulated DEGs and the green font indicates the downregulated DEGs in the left corresponding groups.

To further understand the most important biological processes and molecular functions of the DEGs associated with DNP following STZ treatment, we used gene ontology (GO) analysis to perform enrichment analysis and classifications with the top 20 DEGs from each compared group. Our results showed that, compared the non-DNP with vehicle group, the DEGs were mainly enriched in the “cytosolic ribosome” and involved in “oxygen transport” and “hydrogen peroxide catabolic process”, in which the down-regulated *Hba-a1* and *Hbb* genes were enriched in a variety of molecular functions, including “oxygen binding”,” oxygen carrier activity”,and peroxidase and oxidoreductase activity (Figure 5A). Compared DNP 4w to the non-DNP group, the DEGs were mainly enriched in “receptor ligand activity” and “signaling receptor activator activity”, in which the up-regulated *Ugt1a1*, *Adh7*, and *Aldh1a2* were also enriched in isoprenoid and retinoid binding (Figure 5B). These results suggest that DEGs linked to signaling receptor activation, particularly those mediating rapid isoprenoid and retinoid binding upon STZ exposure, participate in the initial responses to the STZ treatment, including the initiation of induction of DNP induction. Further, in week 8th after STZ treatment, the DEGs were mainly enriched in “extracellular matrix” and “external side of plasma membrane”, which involved “endopeptidase regulator activity, endopeptidase and peptidase inhibitor activity”, and “scavenger receptor activity” (Figure 5C). These results demonstrate strong extracellular signaling transduction and endopeptidase and scavenger receptor activity starting from week 8 after STZ treatment, that were a critical period of time for the development of DNP.

Furthermore, we performed a functional analysis of the top 20 DEGs in diabetes mellitus and pain perception by literature retrieval. The results showed that downregulated DEGs of *Rps6* and *Rpl35a* have been suggested to be associated with diabetes mellitus or pain perception (Gassaway et al., 2018; Wei et al., 2023; Xu et al., 2024), but none of the DEGs were associated with DPN in non-DNP vs. vehicle group (Figure 5D). Compared DNP 4w to the non-DNP group, 9 DEGs were upregulated and 3 were downregulated. Collectively, 83% (10 out of 12) of the DEGs have been reported involved in diabetes mellitus and pain perception, in which *Sema3a* (Aggarwal et al., 2015) *and Cxcl13* (Liu S. et al., 2019) were contribute to advanced diabetic nephropathy and diabetes-induced allodynia respectively (Figure 5D). While, in DNP 8w *vs.* non-DNP group, there are 11 DEGs was upregulated and 16 of them was downregulated, and 56% (15 out of 27) of the DEGs were with known functions in diabetes mellitus or associated with pain perception, and *Mfap4* (Blindbæk et al., 2017), *Kng1* (Tang et al., 2020), *Cd163* (Landis et al., 2018), *Cd180* (Yu and Tao, 2023), and *Csf2rb* (Cao et al., 2022) have been demonstrate play a role in diabetic nephropathy (Figure 5D). These findings suggest important mechanisms underlying the induction and development of DNP and that different transcriptome profiles are involved in the induction and the subsequent development of DNP. Furthermore, these findings remind us that screening DNP-related genes is a necessary and urgent task to understand the pathogenesis of DNP.

### 3.5 Comparison of transcriptomic changes in pain models of nerve injury, bone cancer, and diabetic neuropathy

**FIGURE 6:**
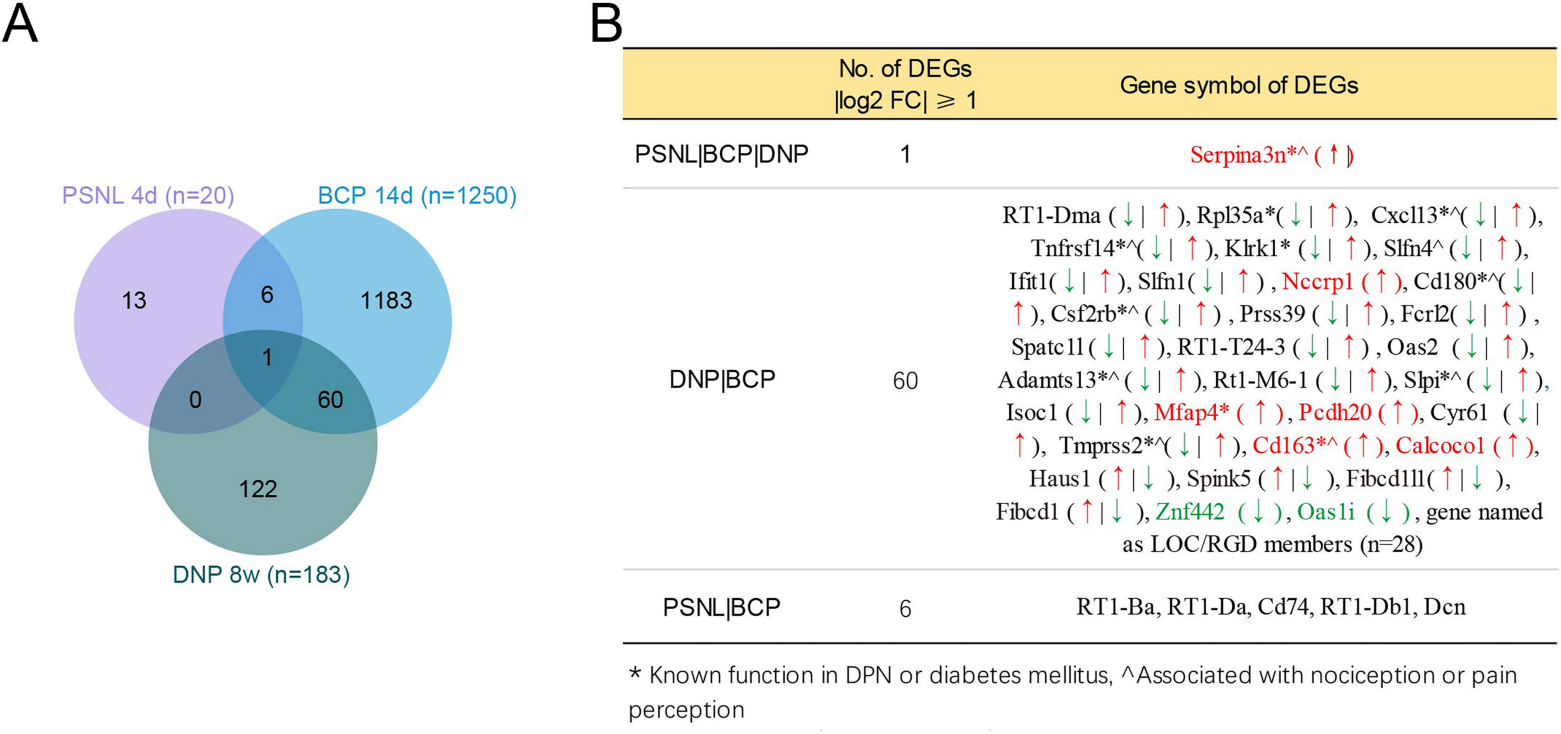
Transcriptomic comparison of the DRG DEGs in rats with different forms of pain each produced by DNP, BCP, and PSNL. **(A)** Venn diagrams showing the numbers of the DEGs that are unique or shared by the three different pain models. **(B)** Function analysis of the shared genes with each comparison that are with known function in DPN or diabetes mellitus or considered associated with nociception or pain perception. The red font highlights the upregulated DEGs and the green font indicates the downregulated DEGs in the left corresponding groups. Up and down arrows indicate the same DEGs were up-regulated and down-regulated respectively in different situations.

The etiology of DNP is intricate, which is considered to be associated with pathological processes such as dysregulation of glucose metabolism, mitochondrial damage, and immune dysregulation. To gain an in-depth understanding of its pathological mechanism and the similarities and differences with other common clinical models, we continued to identify the transcriptomic profiling from models of neuropathic pain, bone cancer pain (BCP), and DNP. Transcriptomic data sets [GSE117526 for partial sciatic nerve ligation (PSNL) (Sun et al., 2020) and GSE149648 for BCP by tumor cells implantation (TCI) (Zhai et al., 2021)] were obtained from Gene Expression Omnibus (GEO) of NCBI. We determined the proportion of DEGs that were unique or shared among the three different pain models. Data were obtained from DRGs taken from rats with TCI (day 14) (Zhai et al., 2021), partial sciatic nerve ligation (PSNL, day 7) (Sun et al., 2020), and DNP (Week 8). The Venn diagrams showed that, in the total DEGs (with |log2 FC| ≥ 1 and normalized *P* value, *q* value ≤ 0.001) from BCP (1250), PSNL (20), and DNP (183), 65–97% DEGs were unique for each of the models, while only 1 gene is shared by the three different models (Figure 6A). As shown in Figure 6B, DEG *Serpina3n* is shared and upregulated in all three models, with known functions in diabetic wound healing (Hsu et al., 2014) and neuropathic pain (Vicuña et al., 2015). There are 60 DEGs shared by DNP and BCP comparable groups. Among these genes, 32 DEGs have highly recognizable symbols (Figure 6B). 31% (10 out of 32) of the DEGs had known functions in diabetes mellitus or were associated with pain perception (Figure 6B). Among these DEGs, *Cxcl13* (Liu S. et al., 2019), *Cd180* (Yu and Tao, 2023), *Csf2rb* (Cao et al., 2022), *Mfap4* (Blindbæk et al., 2017), and *Cd163* (Landis et al., 2018) have been demonstrated to be involved in diabetic nephropathy. Notably, there were 5 and 2 DEGs with consistent up- or down-regulation trends between the two groups, respectively, while 84% (27 out of 32) of DEGs showed opposite trends, suggesting these DEGs may play distinct roles in different pain models depending on the context (Figure 6B). These findings demonstrate that approximately 99% of DEGs are unique and only 1 gene is shared among the three different pain models, suggesting, to a large extent, the heterogeneity of pathogenesis from pain due to DPN, bone cancer, and nerve injury.

## 4 Discussion

The present study reveals the time-course transcriptomic alteration of DRG in a STZ-induced rat model of DPN, integrating behavioral phenotyping, transcriptomic profiling, and cross-model comparisons. The data from the painful group and non-pain group of DPN were differentiated and analyzed. The major findings are Three-fold: (1) the identification of divergent pain phenotypes (DNP vs. non-DNP) within the same STZ-treated rats, highlighting heterogeneity in diabetic neuropathy progression; (2) dynamic transcriptomic changes in DRG revealing stage-specific pathways, including PI3K-Akt signaling and viral infection-related pathways; and (3) the demonstration of distinct transcriptional landscapes between DNP and other pain models, underscoring the unique pathophysiology of DNP. This study reveals the uniqueness of mechanisms underlying DNP, which is, to a large extent, different from the BCP and neuropathic pain, and provides novel potential targets of DEGs for DNP.

### 4.1 Heterogeneity in diabetic neuropathic pain phenotypes

Our observation that only around 60% of STZ-treated rats developed DNP-like behaviors (mechanical allodynia and thermal hyperalgesia) aligns with clinical variability in human diabetic neuropathy, where not all patients progress to painful phenotypes despite similar glycemic profiles (Akinlade et al., 2021; Almomani et al., 2023; Deng et al., 2021). This divergence suggests that hyperglycemia alone is insufficient to drive pain, echoing studies showing that genetic, metabolic, and immune factors modulate neuropathy severity (Almomani et al., 2023). For instance, STZ-induced β-cell destruction triggers systemic oxidative stress and neuroinflammation, which may variably affect sensory neurons depending on individual antioxidant capacity or microglial activation states (Chen and Song, 2024). Notably, the non-DNP group exhibited transcriptomic signatures enriched in cytosolic ribosome and oxygen transport pathways, potentially reflecting compensatory mechanisms against early metabolic stress. These findings parallel human studies linking hemoglobin subunit dysregulation (e.g., Hba1/Hbb) to diabetic complications, suggesting conserved molecular responses across species (Liu et al., 2022; Yapanis et al., 2022). The lack of pain in the cohort of the DPN challenges the traditional view of STZ models as uniformly neuropathic, urging researchers to incorporate phenotypic stratification in preclinical studies. This approach could refine therapeutic targeting, as seen in recent efforts to subtype diabetic neuropathy based on inflammatory biomarkers (Zhang et al., 2024).

Published work has provided evidence for a partial dissociation between cutaneous nerve fiber loss and pain phenotypes in the context of diabetic neuropathy. A clinical study on patients with diabetes found that those with painful distal symmetric polyneuropathy (DSPN) did not differ from those with non-painful DSPN in conventional corneal nerve morphological parameters (e.g., nerve density, length). This suggests that structural nerve damage alone cannot account for differences in pain susceptibility (Sierra-Silvestre et al., 2023). In an STZ-induced mouse model of diabetic neuropathy, early loss of peptidergic intraepidermal nerve fibers was associated with an insensate (pain-free) phenotype, rather than a painful one. This observation demonstrates a clear dissociation between structural nerve fiber loss and the presence of pain (Cheng et al., 2012). Furthermore, the review literature indicates that hyperglycemia-induced nerve damage is the common foundation for both painful and painless diabetic neuropathy. However, differences in pain phenotype arise from abnormal compensatory or hyperexcitable responses to nerve injury (e.g., sodium channel dysregulation, changes in dorsal root ganglion ion channels, aberrant sympathetic sprouting) (Yoo et al., 2013). This implies that structural damage can lead to divergent pain outcomes due to differences in downstream molecular mechanisms. These results strengthen the interpretation that the stage-specific DEGs identified in DRG from the current work are primarily driven by pain-associated neuroimmune remodeling rather than generalized nerve structural damage.

### 4.2 Stage-specific transcriptional dynamics and pathway dysregulation

Our results show that PI3K−Akt and Ras signaling pathways are crucial for the initiation of DNP, while pathways related to virus infection predominantly contribute to the later stages of DNP progression (see Figures 4B,C). The temporal evolution of DRG transcriptomes – from early PI3K-Akt/Ras signaling to late viral infection-related pathways – provides a roadmap for DNP pathogenesis. The PI3K-Akt pathway, a central regulator of neuronal survival and insulin signaling, is hyperactivated in early DNP, consistent with its role in compensating for metabolic stress (Entezari et al., 2022; Liu Y. et al., 2019; Zhang et al., 2021). However, prolonged activation may paradoxically exacerbate neuroinflammation, as observed in diabetic retinopathy models (Chen et al., 2024). Intriguingly, the later enrichment of herpes simplex and Epstein-Barr virus pathways likely reflects the activation of an intrinsic antiviral gene expression program, which may be caused by immune dysregulation or molecular mimicry mechanisms. This hypothesis is supported by clinical associations between diabetes and viral infections, including herpes simplex virus, Epstein-Barr virus, and papillomavirus (Akash et al., 2023; Atiase et al., 2024; Klatka et al., 2023; Kore and Anjankar, 2023; Mahendra et al., 2023; Sundstrom et al., 2024; Wang and Liao, 2022). Notably, recent evidence showing that the activation of transient receptor potential ankyrin 1 (TRPA1) may mediate the calcium ion influx induced by interferon β and the expression of numerous antiviral genes in lung epithelium (Luostarinen et al., 2025). In DPN models, research consistently shows that TRPA1 expression and function in DRG neurons are markedly upregulated and induce neuronal hyperexcitability, pain hypersensitivity, and inflammation (Bautista et al., 2013; Koivisto and Pertovaara, 2013). The channel’s hyperactivity is fueled by endogenous agonists like H_2_O_2_ and reactive carbonyl species (e.g., 4-HNE) generated from diabetic oxidative stress (Andersson et al., 2008). However, direct evidence linking TRPA1 to type I interferon (IFN-I) signaling in diabetic DRG is currently absent. Although neurons possess intrinsic antiviral defenses, including IFN-I production and STAT1 signaling in response to stress, IFN-β is inefficient at protecting primary mouse neurons from virus infection (Delhaye et al., 2006; Kreit et al., 2014). It is plausible that chronic TRPA1 activation in the diabetic milieu acts as a trigger signal. This could potentially trigger a low-grade, IFN-I-like response, contributing to neuroinflammation and interferon-stimulated gene induction within the DRG. Thus, future work investigating this neuroimmune cross-talk could redefine the mechanisms of DPN and provide new insights into the neuroimmune modulation upon TRPA1.

The persistent activation of the CCRI pathway in response to the early induction of STZ treatment, even without pain behavior manifestation (Figure 4A), aligns with our prior work linking chemokine signaling to BCP, which indicated that the immune inflammatory response mediated by the CCRI pathway plays roles in both models (Zhai et al., 2021). Notably, the persistence of coronavirus disease-COVID-19 and other viral infections, including herpes simplex virus, Epstein-Barr virus, and papillomavirus, after the onset of pain behavior is unique in DNP, suggesting that the immune system is dysregulated or impaired in DNP. This hypothesis dovetails with recent interest in re-purposing immune modulator PDA-002 for DPN treatment by a Phase 2a randomized controlled study (Gibbons et al., 2021). Therefore, the observed enrichment in viral infection-related KEGG pathways may signify a generalized state of immune dysregulation and cellular stress response within the DRG microenvironment during chronic DNP.

### 4.3 Unique transcriptomic fingerprints of DNP

The minimal overlap of DEGs between DNP and other pain models (e.g., bone cancer or nerve injury) underscores the specific and distinctive mechanisms between DNP and other pain models (see Figure 6). The shared gene *Serpina3n*, implicated in three different pain models, hints at common inflammatory cascades. The vast majority of DEGs between DNP and BCP were model-specific, and 84% (27 out of 32) of shared DEGs with known gene symbols show opposite regulation in gene expression, indicating the heterogeneity and functional alienation of the same genes in different models and immune environments. For instance, DNP-specific downregulation of Cxcl13 may reflect dysfunction of CXCL13-producing peripheral helper T (Tph) cells, leading to chronic inflammation due to the decline of the ability to suppress inflammation, and neural regeneration disorder (Li et al., 2022; O’Brien et al., 2021; Yoshitomi et al., 2018; Yoshitomi, 2020). Conversely, bone cancer pain models emphasize tumor-nerve crosstalk, highlighting the requirement of tailored therapies for different pain models.

This heterogeneity of the same DEGs in different models resonates with clinical data showing that DNP patients respond poorly to gabapentinoids effective in post-herpetic neuralgia (Menaldi et al., 2022). Our findings thus advocate for mechanism-based stratification of neuropathic pain, as proposed in recent precision medicine initiatives.

It is important to note that the time points analyzed differ across models (DNP: 8 weeks; BCP: 14 days; PSNL: 4 days), primarily reflecting the characteristic progression timelines of each etiology. While this design captures each model at a well-established pain maintenance phase, we acknowledge that some observed differences could be attributable to disease stage dynamics in addition to etiology-specific mechanisms. Future studies comparing models at equivalent pathophysiological stages would be valuable to further disentangle these factors.

### 4.4 Therapeutic implications and future directions

The dynamic pathway alterations identified in the current study suggest time-sensitive therapeutic windows. Early targeting of PI3K-Akt signaling – e.g., with metformin or isoform-specific inhibitors – may mitigate neuroinflammation before irreversible damage occurs. Conversely, late-stage interventions could focus on viral mimicry pathways or extracellular matrix remodeling, leveraging antiviral agents or MMP inhibitors (Baldimtsi et al., 2023; Papadopoulou-Marketou et al., 2021; Yadav, 2023).

Furthermore, the dysregulation of 5-HT-related pathways (implied by neuroactive ligand-receptor enrichment) invites exploration of serotonergic modulators, which have shown promise in diabetic β-cell regeneration (Liu N. et al., 2025). Notably, the shared gene Cd163 – a marker of anti-inflammatory macrophages – highlights the potential of immunomodulatory strategies to restore nerve homeostasis (Hakim et al., 2025).

### 4.5 Limitations and future perspectives

Our study has several limitations. First, the use of bulk RNA-seq, while providing a comprehensive overview, cannot resolve cell type-specific contributions to the observed transcriptomic changes. Given the known infiltration of immune cells and altered neuron-glial interactions in DPN, future single-cell or spatial transcriptomic studies are warranted to pinpoint the cellular origins of key pathways. Second, our study was conducted exclusively in male rats. Given the established sex differences in pain processing and immune responses, investigating female subjects in future work is crucial for translational relevance. Third, while the STZ-induced type 1 diabetes model is widely used, it does not fully replicate the slow progression and profound neurodegenerative features (e.g., Nageotte nodules) observed in human diabetic DRG over the years. This discrepancy should be considered when extrapolating findings to human DPN. Finally, recent transcriptomic profiling of human DRG from diabetic patients with and without pain revealed dysregulation in pathways like oxidative phosphorylation, mTORC1 signaling, TNFa/NF-KB signaling, p53 pathway, interferon response, apoptosis, unfolded protein response, and complement (Ferreira et al., 2024). These results show limited overlap with the late-stage transcriptomic profile observed in STZ-induced rat DNP in the current study. Despite these discrepancies that may arise from species, disease stage differences, and transcriptomic analysis sensitivity, a notable convergence is oxidative phosphorylation and the dysregulation of interferon-response- and antiviral-defense-associated genes in human and rat datasets, respectively. This supports one of our central hypotheses that immune dysregulation, particularly the aberrant activation of innate immune/antiviral defense pathways, is a conserved, cross-species feature of DNP. Therefore, these findings reveal the key role of sustained cellular stress and neuro-immune crosstalk in DNP pathophysiology. Future studies are needed to validate and integrate these cross-species discoveries, distinguishing pain-specific core mechanisms of DNP. In our subsequent independent experiments, we will systematically compare the degree of cutaneous nerve loss with pain behavioral scores and DRG transcriptomic signatures to disentangle transcriptional changes specific to nerve degeneration versus pain sensitization. Furthermore, we will adopt RNAscope *in situ* hybridization combined with cell-type-specific marker immunostaining to precisely localize the key DEGs identified in this study to distinct DRG cellular subpopulations. We will further quantify cell-specific expression differences between DNP and non-DNP rats to disentangle neuron-, glia-, and macrophage-derived transcriptional signals, which will provide direct cell-level evidence to support the stage-specific pathogenic pathways uncovered by bulk transcriptomics.

## 5 Conclusion

This study delineates the molecular choreography of DNP progression, offering a framework for stage-specific interventions. By integrating phenotypic heterogeneity, dynamic transcriptomics, and cross-model comparisons, we advance the mechanistic understanding of diabetic neuropathy beyond glycemic control. Future work should validate these pathways in human tissues and explore their utility in personalized pain management. Our findings may not only refine existing pathophysiological models but also catalyze the development of precision therapies targeting DRG-specific pathways.

## Data Availability

The datasets presented in this study can be found in online repositories. The names of the repository/repositories and accession number(s) can be found in the article/Supplementary material.

## References

[B1] Abd Razak N. H. Idris J. Hassan N. H. Zaini F. Muhamad N. Daud M. F. (2024). Unveiling the role of schwann cell plasticity in the pathogenesis of diabetic peripheral neuropathy. *Int. J. Mol. Sci.* 25:10785. 10.3390/ijms251910785 39409114 PMC11476695

[B2] Aggarwal P. Veron D. Thomas D. Siegel D. Moeckel G. Kashgarian M. et al.. (2015). Semaphorin3a promotes advanced diabetic nephropathy. *Diabetes* 64 1743–1759. 10.2337/db14-0719 25475434 PMC4407856

[B3] Akash S. Azad M. Mukerjee N. Bibi S. (2023). Re-emergence of herpes simplex virus type-1 and type-2 manifestations in type 2 diabetes patients: Its current status, genomic characteristics, diagnosis, treatment, and future outlook. *Int. J. Surg.* 109 96–98. 10.1097/JS9.0000000000000245 36799815 PMC10389202

[B4] Akinlade O. M. Owoyele B. V. Soladoye A. O. (2021). Streptozotocin-induced type 1 and 2 diabetes in rodents: A model for studying diabetic cardiac autonomic neuropathy. *Afr. Health Sci.* 21 719–727. 10.4314/ahs.v21i2.30 34795728 PMC8568204

[B5] Almomani R. Sopacua M. Marchi M. Ślęczkowska M. Lindsey P. de Greef B. et al.. (2023). Genetic profiling of sodium channels in diabetic painful and painless and idiopathic painful and painless neuropathies. *Int. J. Mol. Sci.* 24:8278. 10.3390/ijms24098278 37175987 PMC10179245

[B6] Andersson D. Gentry C. Moss S. Bevan S. (2008). Transient receptor potential A1 is a sensory receptor for multiple products of oxidative stress. *J. Neurosci.* 28 2485–2494. 10.1523/JNEUROSCI.5369-07.2008 18322093 PMC2709206

[B7] Athie M. Vieira A. Teixeira J. Dos Santos G. Dias E. Tambeli C. et al.. (2018). Transcriptome analysis of dorsal root ganglia’s diabetic neuropathy reveals mechanisms involved in pain and regeneration. *Life Sci.* 205 54–62. 10.1016/j.lfs.2018.05.016 29750992

[B8] Atiase Y. Effah K. Mawusi Wormenor C. Tekpor E. Aku Catherine Morkli E. Boafo E. et al.. (2024). Prevalence of high-risk human papillomavirus infection among women with diabetes mellitus in Accra, Ghana. *BMC Womens Health* 24:260. 10.1186/s12905-024-03078-z 38664791 PMC11044360

[B9] Baldimtsi E. Whiss P. Wahlberg J. (2023). Systemic biomarkers of microvascular alterations in type 1 diabetes associated neuropathy and nephropathy - A prospective long-term follow-up study. *J. Diabetes Complications* 37:108635. 10.1016/j.jdiacomp.2023.108635 37989066

[B10] Baral P. Umans B. Li L. Wallrapp A. Bist M. Kirschbaum T. et al.. (2018). Nociceptor sensory neurons suppress neutrophil and γδ T cell responses in bacterial lung infections and lethal pneumonia. *Nat. Med.* 24 417–426. 10.1038/nm.4501 29505031 PMC6263165

[B11] Baum P. Toyka K. Blüher M. Kosacka J. Nowicki M. (2021). Inflammatory mechanisms in the pathophysiology of diabetic peripheral neuropathy (DN)-new aspects. *Int J. Mol. Sci.* 22:10835. 10.3390/ijms221910835 34639176 PMC8509236

[B12] Bautista D. Pellegrino M. Tsunozaki M. (2013). TRPA1: A gatekeeper for inflammation. *Annu. Rev. Physiol.* 75 181–200. 10.1146/annurev-physiol-030212-183811 23020579 PMC4041114

[B13] Blindbæk S. Schlosser A. Green A. Holmskov U. Sorensen G. Grauslund J. (2017). Association between microfibrillar-associated protein 4 (MFAP4) and micro- and macrovascular complications in long-term type 1 diabetes mellitus. *Acta Diabetol.* 54 367–372. 10.1007/s00592-016-0953-y 28039584

[B14] Bowling F. Rashid S. Boulton A. (2015). Preventing and treating foot complications associated with diabetes mellitus. *Nat. Rev. Endocrinol.* 11 606–616. 10.1038/nrendo.2015.130 26284447

[B15] Calcutt N. (2020). Diabetic neuropathy and neuropathic pain: A (con)fusion of pathogenic mechanisms? *Pain* 161 S65–S86. 10.1097/j.pain.0000000000001922 32999525 PMC7521457

[B16] Cao H. Rao X. Jia J. Yan T. Li D. (2022). Identification of tubulointerstitial genes and ceRNA networks involved in diabetic nephropathy via integrated bioinformatics approaches. *Hereditas* 159:36. 10.1186/s41065-022-00249-6 36154667 PMC9511769

[B17] Chaplan S. Bach F. Pogrel J. Chung J. Yaksh T. (1994). Quantitative assessment of tactile allodynia in the rat paw. *J. Neurosci. Methods* 53 55–63. 10.1016/0165-0270(94)90144-9 7990513

[B18] Chen L. Zhang H. Zhang Y. Li X. Wang M. Shen Y. et al.. (2024). Ganglion cell-derived LysoPS induces retinal neovascularisation by activating the microglial GPR34-PI3K-AKT-NINJ1 axis. *J. Neuroinflammation* 21:278. 10.1186/s12974-024-03265-7 39468551 PMC11520652

[B19] Chen Y. Song X. (2024). Diabetic neuropathic pain: Directions for exploring treatments. *Biomedicines* 12:589. 10.3390/biomedicines12030589 38540203 PMC10967923

[B20] Cheng H. Dauch J. Hayes J. Yanik B. Feldman E. (2012). Nerve growth factor/p38 signaling increases intraepidermal nerve fiber densities in painful neuropathy of type 2 diabetes. *Neurobiol. Dis.* 45 280–287. 10.1016/j.nbd.2011.08.011 21872660 PMC3225563

[B21] Delhaye S. Paul S. Blakqori G. Minet M. Weber F. Staeheli P. et al.. (2006). Neurons produce type I interferon during viral encephalitis. *Proc. Natl. Acad. Sci. U S A.* 103 7835–7840. 10.1073/pnas.0602460103 16682623 PMC1458506

[B22] Deng X. Ma P. Wu M. Liao H. Song X. (2021). Role of matrix metalloproteinases in myelin abnormalities and mechanical allodynia in rodents with diabetic neuropathy. *Aging Dis.* 12 1808–1820. 10.14336/AD.2021.0126 34631222 PMC8460301

[B23] Du H. Liu Z. Tan X. Ma Y. Gong Q. (2019). Identification of the genome-wide expression patterns of long non-coding RNAs and mRNAs in mice with streptozotocin-induced diabetic neuropathic pain. *Neuroscience* 402 90–103. 10.1016/j.neuroscience.2018.12.040 30599267

[B24] Eid S. Elzinga S. Guo K. Hinder L. Hayes J. Pacut C. et al.. (2024). Transcriptomic profiling of sciatic nerves and dorsal root ganglia reveals site-specific effects of prediabetic neuropathy. *Transl. Res.* 270 24–41. 10.1016/j.trsl.2024.03.009 38556110 PMC11166517

[B25] Eid S. Rumora A. Beirowski B. Bennett D. Hur J. Savelieff M. et al.. (2023). New perspectives in diabetic neuropathy. *Neuron* 111 2623–2641. 10.1016/j.neuron.2023.05.003 37263266 PMC10525009

[B26] Elafros M. Andersen H. Bennett D. Savelieff M. Viswanathan V. Callaghan B. et al.. (2022). Towards prevention of diabetic peripheral neuropathy: Clinical presentation, pathogenesis, and new treatments. *Lancet Neurol.* 21 922–936. 10.1016/S1474-4422(22)00188-0 36115364 PMC10112836

[B27] Entezari M. Hashemi D. Taheriazam A. Zabolian A. Mohammadi S. Fakhri F. et al.. (2022). AMPK signaling in diabetes mellitus, insulin resistance and diabetic complications: A pre-clinical and clinical investigation. *Biomed. Pharmacother.* 146:112563. 10.1016/j.biopha.2021.112563 35062059

[B28] Ferreira D. T. Shen B. Q. Mwirigi J. M. Shiers S. Sankaranarayanan I. Kotamarti M. et al.. (2024). Deciphering the molecular landscape of human peripheral nerves: Implications for diabetic peripheral neuropathy. *bioRxiv* [Preprint] 10.1101/2024.06.15.599167 38915676 PMC11195245

[B29] Gassaway B. M. Petersen M. C. Surovtseva Y. V. Barber K. W. Sheetz J. B. Aerni H. R. et al.. (2018). PKCepsilon contributes to lipid-induced insulin resistance through cross talk with p70S6K and through previously unknown regulators of insulin signaling. *Proc. Natl. Acad. Sci. U S A.* 115 E8996–E9005. 10.1073/pnas.1804379115 30181290 PMC6156646

[B30] GBD 2021 Forecasting Collaborators. (2024). Forecasting Burden of disease scenarios for 204 countries and territories, 2022-2050: A forecasting analysis for the Global Burden of Disease Study 2021. *Lancet* 403 2204–2056. 10.1016/S0140-6736(24)00685-8 38762325 PMC11121021

[B31] Gibbons C. Zhu J. Zhang X. Habboubi N. Hariri R. Veves A. (2021). Phase 2a randomized controlled study investigating the safety and efficacy of PDA-002 in diabetic peripheral neuropathy. *J. Peripher. Nerv. Syst.* 26 276–289. 10.1111/jns.12457 34169613

[B32] Guo G. Chen J. Shen Q. Chen Z. (2024). Single-cell transcriptome analysis reveals distinct cell populations in dorsal root ganglia and their potential roles in diabetic peripheral neuropathy. *PLoS One* 19:e0306424. 10.1371/journal.pone.0306424 39083491 PMC11290642

[B33] Hakim S. Jain A. Adamson S. Petrova V. Indajang J. Kim H. et al.. (2025). Macrophages protect against sensory axon loss in peripheral neuropathy. *Nature* 640 212–220. 10.1038/s41586-024-08535-1 39939762 PMC11964918

[B34] Hall B. Macdonald E. Cassidy M. Yun S. Sapio M. Ray P. et al.. (2022). Transcriptomic analysis of human sensory neurons in painful diabetic neuropathy reveals inflammation and neuronal loss. *Sci. Rep.* 12:4729. 10.1038/s41598-022-08100-8 35304484 PMC8933403

[B35] Hanč P. Gonzalez R. Mazo I. Wang Y. Lambert T. Ortiz G. et al.. (2023). Multimodal control of dendritic cell functions by nociceptors. *Science* 379:eabm5658. 10.1126/science.abm5658 36996219 PMC10642951

[B36] Hinder L. Murdock B. Park M. Bender D. O’Brien P. Rumora A. et al.. (2018). Transcriptional networks of progressive diabetic peripheral neuropathy in the db/db mouse model of type 2 diabetes: An inflammatory story. *Exp. Neurol.* 305 33–43. 10.1016/j.expneurol.2018.03.011 29550371 PMC5955815

[B37] Hsu I. Parkinson L. Shen Y. Toro A. Brown T. Zhao H. et al.. (2014). Serpina3n accelerates tissue repair in a diabetic mouse model of delayed wound healing. *Cell. Death Dis.* 5:e1458. 10.1038/cddis.2014.423 25299783 PMC4237249

[B38] Kanehisa M. (2019). Toward understanding the origin and evolution of cellular organisms. *Protein Sci.* 28 1947–1951. 10.1002/pro.3715 31441146 PMC6798127

[B39] Kanehisa M. Goto S. (2000). KEGG: Kyoto encyclopedia of genes and genomes. *Nucleic Acids Res.* 28 27–30. 10.1093/nar/28.1.27 10592173 PMC102409

[B40] Kanehisa M. Furumichi M. Sato Y. Matsuura Y. Ishiguro-Watanabe M. (2025). KEGG: Biological systems database as a model of the real world. *Nucleic Acids Res.* 53 D672–D677. 10.1093/nar/gkae909 39417505 PMC11701520

[B41] Klatka M. Rysz I. Hymos A. Polak A. Mertowska P. Mertowski S. et al.. (2023). Effect of epstein-barr virus infection on selected immunological parameters in children with type 1 diabetes. *Int. J. Mol. Sci.* 24:2392. 10.3390/ijms24032392 36768715 PMC9917181

[B42] Koivisto A. Pertovaara A. (2013). Transient receptor potential ankyrin 1 (TRPA1) ion channel in the pathophysiology of peripheral diabetic neuropathy. *Scand. J. Pain* 4 129–136. 10.1016/j.sjpain.2012.11.001 29913916

[B43] Kore V. B. Anjankar A. A. (2023). Comprehensive review of treatment approaches for cutaneous and genital warts. *Cureus* 15:e47685. 10.7759/cureus.47685 38022045 PMC10673707

[B44] Kreit M. Paul S. Knoops L. De Cock A. Sorgeloos F. Michiels T. (2014). Inefficient type I interferon-mediated antiviral protection of primary mouse neurons is associated with the lack of apolipoprotein l9 expression. *J. Virol.* 88 3874–3884. 10.1128/JVI.03018-13 24453359 PMC3993554

[B45] Landis R. Quimby K. Greenidge A. (2018). M1/M2 macrophages in diabetic nephropathy: Nrf2/HO-1 as therapeutic targets. *Curr. Pharm. Des.* 24 2241–2249. 10.2174/1381612824666180716163845 30014796

[B46] Li B. Dewey C. N. (2011). RSEM: Accurate transcript quantification from RNA-Seq data with or without a reference genome. *BMC Bioinformatics* 12:323. 10.1186/1471-2105-12-323 21816040 PMC3163565

[B47] Li W. Guo J. Chen J. Yao H. Mao R. Li C. et al.. (2022). Identification of immune infiltration and the potential biomarkers in diabetic peripheral neuropathy through bioinformatics and machine learning methods. *Biomolecules* 13:39. 10.3390/biom13010039 36671424 PMC9855866

[B48] Liu C. Chen H. Ma Y. Zhang L. Chen L. Huang J. et al.. (2025). Clinical metabolomics in type 2 diabetes mellitus: From pathogenesis to biomarkers. *Front. Endocrinol.* 16:1501305. 10.3389/fendo.2025.1501305 40070584 PMC11893406

[B49] Liu N. Liu T. Alim N. Zou J. Hu X. Zhang B. et al.. (2025). 5-HT promotes pancreatic α-to-β cell transdifferentiation. *Biochim. Biophys. Acta Mol. Cell. Res.* 1872:119958. 10.1016/j.bbamcr.2025.119958 40222656

[B50] Liu S. Liu X. Xiong H. Wang W. Liu Y. Yin L. et al.. (2019). CXCL13/CXCR5 signaling contributes to diabetes-induced tactile allodynia via activating pERK, pSTAT3, pAKT pathways and pro-inflammatory cytokines production in the spinal cord of male mice. *Brain Behav. Immun.* 80 711–724. 10.1016/j.bbi.2019.05.020 31100371

[B51] Liu Y. Liu B. Qiao Y. Niu W. Y. (2022). A new case of Hb headington (HBB: c.217A>C) due to a new DNA transversion, found in a patient with type 2 diabetes mellitus. *Hemoglobin* 46 180–183. 10.1080/03630269.2022.2067044 35603587

[B52] Liu Y. Zhang S. Xue J. Wei Z. Ao P. Shen B. et al.. (2019). CGRP reduces apoptosis of DRG cells induced by high-glucose oxidative stress injury through PI3K/AKT induction of heme oxygenase-1 and Nrf-2 expression. *Oxid. Med. Cell. Longev.* 2019:2053149. 10.1155/2019/2053149 31885775 PMC6899316

[B53] Lu Y. Nayer B. Singh S. Alshoubaki Y. Yuan E. Park A. et al.. (2024). CGRP sensory neurons promote tissue healing via neutrophils and macrophages. *Nature* 628 604–611. 10.1038/s41586-024-07237-y 38538784 PMC11023938

[B54] Luostarinen S. Pemmari A. Vistbacka J. Sioofy-Khojine A. Hämäläinen M. Hyöty H. et al.. (2025). Transient receptor potential ankyrin 1 promotes the expression of interferon-stimulated antiviral genes in human A549 lung epithelial cells. *Mol. Pharmacol.* 108:100098. 10.1016/j.molpha.2025.100098 41539023 PMC12975360

[B55] Mahendra J. Mahendra L. Divya D. Ilango P. Devarajan N. Thanigaimalai A. (2023). Association of Epstein-Barr virus, cytomegalovirus and lipocalin with periodontitis in type 2 diabetic subjects. *Oral. Dis.* 29 1163–1171. 10.1111/odi.14091 34850506

[B56] Menaldi S. L. Halim P. A. Kurniawan K. (2022). Efficacy of gabapentinoids for acute herpes zoster in preventing postherpetic neuralgia: A systematic review of randomized controlled trials. *Dermatol. Online J.* 28. 10.5070/D328559238 36809125

[B57] O’Brien J. McGuire H. Shinko D. Fazekas de St Groth B. Russo M. A. Bailey D. et al.. (2021). T lymphocyte and monocyte subsets are dysregulated in type 1 diabetes patients with peripheral neuropathic pain. *Brain Behav. Immun. Health* 15:100283. 10.1016/j.bbih.2021.100283 34589782 PMC8474166

[B58] Pacifico P. George D. Jayaraj N. D. Ren D. Coy-Dibley J. S. Belmadani A. A. et al.. (2026). Skin-resident Langerhans cells drive neuropathic pain via chemokine-dependent neuron-immune communication. *J. Clin. Invest.* 136:e192328. 10.1172/JCI192328 42060359 PMC13262737

[B59] Papadopoulou-Marketou N. Whiss P. Eriksson A. Hyllienmark L. Papassotiriou I. Wahlberg J. (2021). Plasma levels of tissue inhibitor of metalloproteinase-1 in patients with type 1 diabetes mellitus associate with early diabetic neuropathy and nephropathy. *Diab. Vasc. Dis. Res.* 18:14791641211002470. 10.1177/14791641211002470 33775157 PMC8481743

[B60] Sierra-Silvestre E. Andrade R. Colorado L. Edwards K. Coppieters M. (2023). Occurrence of corneal sub-epithelial microneuromas and axonal swelling in people with diabetes with and without (painful) diabetic neuropathy. *Diabetologia* 66 1719–1734. 10.1007/s00125-023-05945-0 37301795 PMC10257488

[B61] Smith D. Anderson D. Degryse A. Bol C. Criado A. Ferrara A. et al.. (2018). Classification and reporting of severity experienced by animals used in scientific procedures: FELASA/ECLAM/ESLAV working group report. *Lab. Anim.* 52 5–57. 10.1177/0023677217744587 29359995 PMC5987990

[B62] Sun W. Kou D. Yu Z. Yang S. Jiang C. Xiong D. et al.. (2020). A transcriptomic analysis of neuropathic pain in rat dorsal root ganglia following peripheral nerve injury. *Neuromol. Med.* 22 250–263. 10.1007/s12017-019-08581-3 31858405

[B63] Sun W. Li C. Zhang Y. Jiang C. Zhai M. Zhou Q. et al.. (2017). Gene expression changes of thermo-sensitive transient receptor potential channels in obese mice. *Cell. Biol. Int.* 41 908–913. 10.1002/cbin.10783 28464448

[B64] Sundstrom J. Vanderleeden E. Barton N. Redick S. Dawes P. Murray L. et al.. (2024). Herpes simplex virus 1 infection of human brain organoids and pancreatic stem cell-islets drives organoid-specific transcripts associated with Alzheimer’s disease and autoimmune diseases. *Cells* 13:1978. 10.3390/cells13231978 39682726 PMC11640215

[B65] Tang S. Wang X. Deng T. Ge H. Xiao X. (2020). Identification of C3 as a therapeutic target for diabetic nephropathy by bioinformatics analysis. *Sci. Rep.* 10:13468. 10.1038/s41598-020-70540-x 32778679 PMC7417539

[B66] Vicuña L. Strochlic D. Latremoliere A. Bali K. Simonetti M. Husainie D. et al.. (2015). The serine protease inhibitor SerpinA3N attenuates neuropathic pain by inhibiting T cell-derived leukocyte elastase. *Nat. Med.* 21 518–523. 10.1038/nm.3852 25915831 PMC4450999

[B67] Wang S. Liao J. (2022). Epidemiologic implication of the association between herpes simplex virus infection and the risk of type 1 diabetes mellitus: A nationwide case-control study in Taiwan. *Int. J. Environ. Res. Public Health* 19:7832. 10.3390/ijerph19137832 35805493 PMC9265894

[B68] Wei W. Zhang Q. Jin T. Zhu L. Zhao J. Li F. et al.. (2023). Quantitative proteomics characterization of the effect and mechanism of trichostatin A on the hippocampus of type II diabetic mice. *Cell. Mol. Neurobiol.* 43 4309–4332. 10.1007/s10571-023-01424-7 37864628 PMC11407725

[B69] Xu F. Chen A. Pan S. Wu Y. He H. Han Z. et al.. (2024). Systems genetics analysis reveals the common genetic basis for pain sensitivity and cognitive function. *CNS Neurosci. Ther.* 30:e14557. 10.1111/cns.14557 38421132 PMC10850811

[B70] Yadav J. P. (2023). Based on clinical research Matrix metalloprotease (MMP) inhibitors to promote diabetic wound healing. *Horm. Metab. Res.* 55 752–757. 10.1055/a-2171-5879 37798905

[B71] Yang D. Jacobson A. Meerschaert K. Sifakis J. Wu M. Chen X. et al.. (2022). Nociceptor neurons direct goblet cells via a CGRP-RAMP1 axis to drive mucus production and gut barrier protection. *Cell* 185 4190–4205.e25. 10.1016/j.cell.2022.09.024 36243004 PMC9617795

[B72] Yao X. Wang X. Zhang R. Kong L. Fan C. Qian Y. (2025). Dysregulated mast cell activation induced by diabetic milieu exacerbates the progression of diabetic peripheral neuropathy in mice. *Nat. Commun.* 16:4170. 10.1038/s41467-025-59562-z 40325050 PMC12052842

[B73] Yapanis M. James S. Craig M. O’Neal D. Ekinci E. (2022). Complications of diabetes and metrics of glycemic management derived from continuous glucose monitoring. *J. Clin. Endocrinol. Metab.* 107 e2221–e2236. 10.1210/clinem/dgac034 35094087 PMC9113815

[B74] Ye L. Huang H. Zhang S. Lu J. Cao D. Wu D. et al.. (2021). Streptozotocin-induced hyperglycemia affects the pharmacokinetics of koumine and its anti-allodynic action in a rat model of diabetic neuropathic pain. *Front. Pharmacol.* 12:640318. 10.3389/fphar.2021.640318 34054521 PMC8156416

[B75] Yoo M. Sharma N. Pasnoor M. Kluding P. M. (2013). Painful diabetic peripheral neuropathy: Presentations, mechanisms, and exercise therapy. *J. Diabetes Metab.* 10(Suppl.):005. 10.4172/2155-6156.S10-005 25360348 PMC4211105

[B76] Yoshitomi H. (2020). CXCL13-producing PD-1(hi)CXCR5(-) helper T cells in chronic inflammation. *Immunol. Med.* 43 156–160. 10.1080/25785826.2020.1781998 32584200

[B77] Yoshitomi H. Kobayashi S. Miyagawa-Hayashino A. Okahata A. Doi K. Nishitani K. et al.. (2018). Human Sox4 facilitates the development of CXCL13-producing helper T cells in inflammatory environments. *Nat. Commun.* 9:3762. 10.1038/s41467-018-06187-0 30232328 PMC6145936

[B78] Yu G. Wang L. Han Y. He Q. (2012). clusterProfiler: An R package for comparing biological themes among gene clusters. *OMICS* 16 284–287. 10.1089/omi.2011.0118 22455463 PMC3339379

[B79] Yu L. Tao H. (2023). Screening of diabetic nephropathy progression-related genes based on weighted gene co-expression network analysis. *Biochem. Genet.* 61 221–237. 10.1007/s10528-022-10250-3 35834115

[B80] Zhai M. Huang J. Yang S. Li N. Zeng J. Zheng Y. et al.. (2024). Transcriptomic analysis of differentially alternative splicing patterns in mice with inflammatory and neuropathic pain. *Mol. Pain* 20:17448069241249455. 10.1177/17448069241249455 38597175 PMC11084985

[B81] Zhai M. Yang S. Lin S. Zhu H. Xu L. Liao H. et al.. (2021). Distinct gene expression patterns of ion channels and cytokines in rat primary sensory neurons during development of bone cancer and cancer pain. *Front. Mol. Neurosci.* 14:665085. 10.3389/fnmol.2021.665085 34025351 PMC8134751

[B82] Zhang P. Wang C. Li C. Wang J. (2024). miR-34a-5p predicts the risk of diabetic neuropathic pain and mediates neuroinflammation in microglia via targeting ENPP3. *Immunol. Invest.* 53 1348–1358. 10.1080/08820139.2024.2400550 39252196

[B83] Zhang X. Zhao S. Yuan Q. Zhu L. Li F. Wang H. et al.. (2021). TXNIP, a novel key factor to cause Schwann cell dysfunction in diabetic peripheral neuropathy, under the regulation of PI3K/Akt pathway inhibition-induced DNMT1 and DNMT3a overexpression. *Cell. Death Dis.* 12:642. 10.1038/s41419-021-03930-2 34162834 PMC8222353

[B84] Zhang Y. Huang Z. Liu S. Liu Y. Song A. Song X. J. (2013). WNT signaling underlies the pathogenesis of neuropathic pain in rodents. *J. Clin. Invest.* 123 2268–2286. 10.1172/JCI65364 23585476 PMC3635721

[B85] Zhou H. Yang X. Liao C. Chen H. Wu Y. Xie B. et al.. (2022). The development of mechanical allodynia in diabetic rats revealed by single-cell RNA-Seq. *Front. Mol. Neurosci.* 15:856299. 10.3389/fnmol.2022.856299 35668789 PMC9165721

