# Supplementary material for: Dynamic transcriptomic profiling of dorsal root ganglia reveals stage-specific mechanisms in diabetic neuropathic pain
Source: Front Mol Neurosci. 2026 Aug 5;19:1870499. doi: 10.3389/fnmol.2026.1870499 (PMC13486286; doi:10.3389/fnmol.2026.1870499)
Supplement: Supplementary file 1 [file Image_1.pdf]

Figure S1

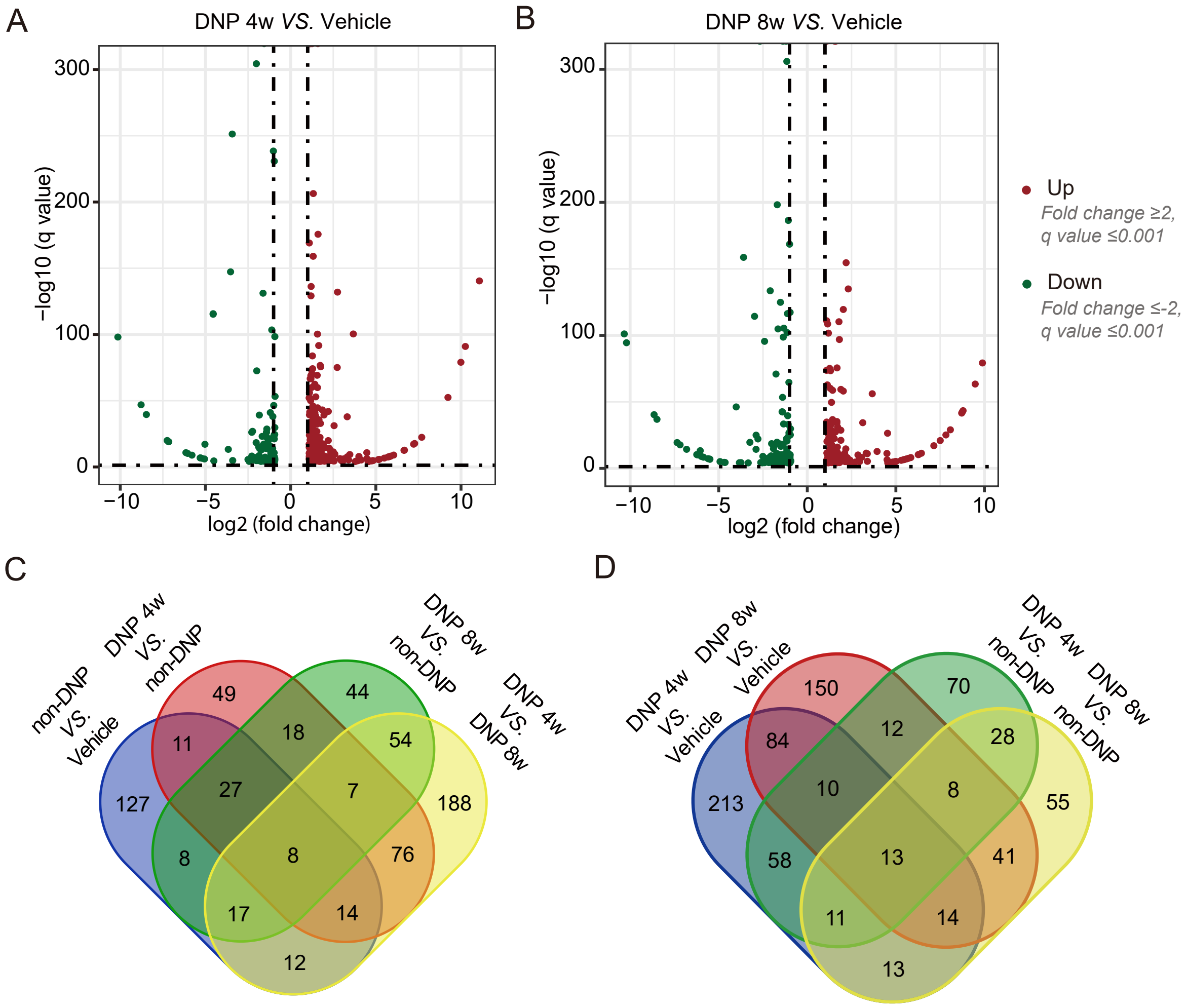

A
DNP 4w VS. Vehicle
−log10 (q value)
log2 (fold change)
B
DNP 8w VS. Vehicle
−log10 (q value)
log2 (fold change)
Up
Fold change ≥2,
q value ≤0.001
Down
Fold change ≤-2,
q value ≤0.001
C
non-DNP VS. Vehicle
DNP 4w VS. non-DNP
DNP 8w VS. non-DNP
DNP 4w VS. DNP 8w
127
49
44
188
11
18
54
27
7
8
8
76
17
14
12
D
DNP 4w VS. Vehicle
DNP 8w VS. Vehicle
DNP 4w VS. non-DNP
DNP 8w VS. non-DNP
213
150
70
55
84
12
28
10
8
58
13
41
11
14
13
