# Supplementary material for: Dynamic transcriptomic profiling of dorsal root ganglia reveals stage-specific mechanisms in diabetic neuropathic pain
Source: Front Mol Neurosci. 2026 Aug 5;19:1870499. doi: 10.3389/fnmol.2026.1870499 (PMC13486286; doi:10.3389/fnmol.2026.1870499)
Supplement: Supplementary file 2 [file Image_2.pdf]

# Figure S2

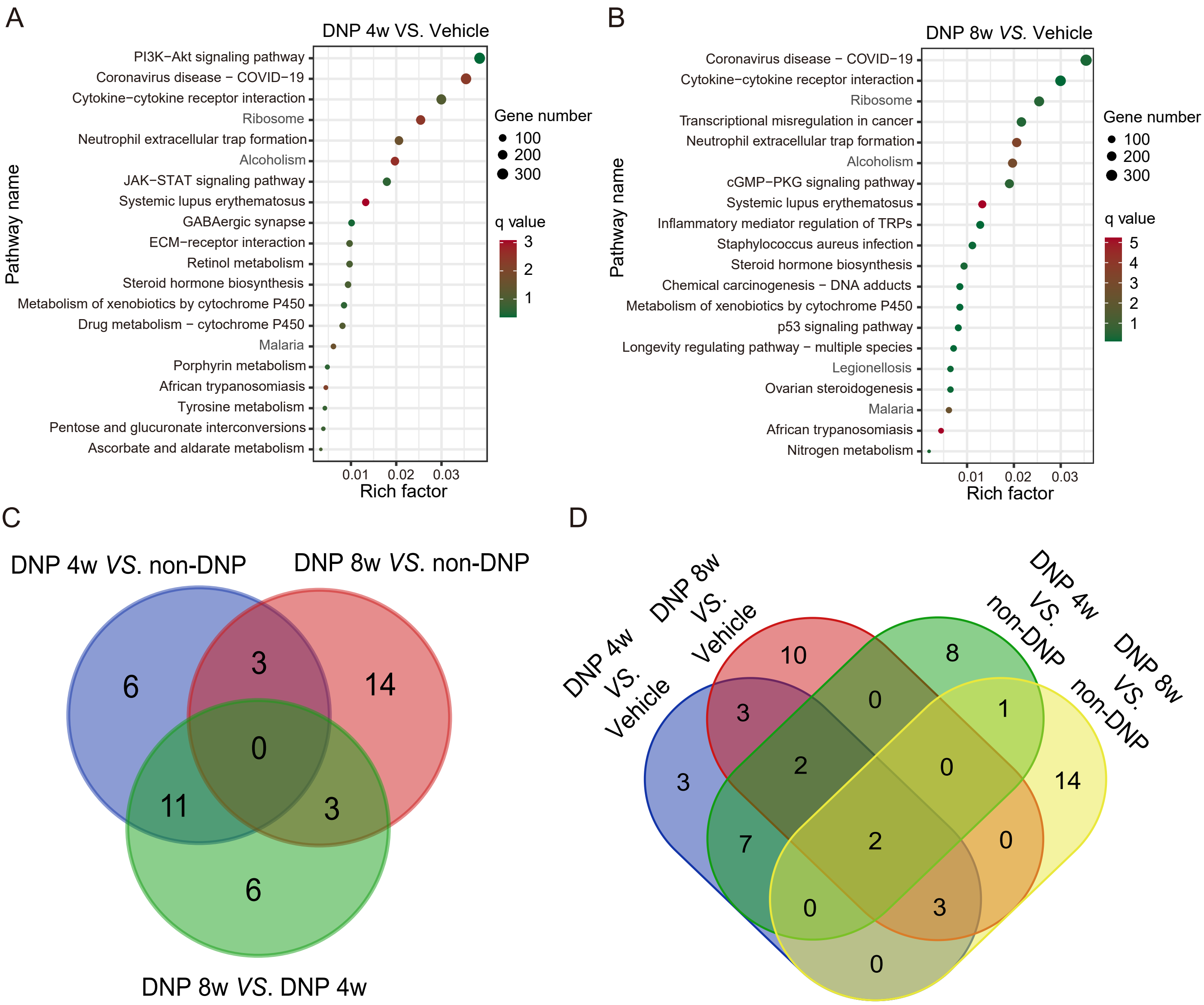
A
DNP 4w VS. Vehicle
PI3K−Akt signaling pathway
Coronavirus disease − COVID−19
Cytokine−cytokine receptor interaction
Ribosome
Neutrophil extracellular trap formation
Alcoholism
JAK−STAT signaling pathway
Systemic lupus erythematosus
GABAergic synapse
ECM−receptor interaction
Retinol metabolism
Steroid hormone biosynthesis
Metabolism of xenobiotics by cytochrome P450
Drug metabolism − cytochrome P450
Malaria
Porphyrin metabolism
African trypanosomiasis
Tyrosine metabolism
Pentose and glucuronate interconversions
Ascorbate and aldarate metabolism
Pathway name
Rich factor
0.01 0.02 0.03
Gene number
100
200
300
q value
3
2
1
B
DNP 8w VS. Vehicle
Coronavirus disease − COVID−19
Cytokine−cytokine receptor interaction
Ribosome
Transcriptional misregulation in cancer
Neutrophil extracellular trap formation
Alcoholism
cGMP−PKG signaling pathway
Systemic lupus erythematosus
Inflammatory mediator regulation of TRPs
Staphylococcus aureus infection
Steroid hormone biosynthesis
Chemical carcinogenesis − DNA adducts
Metabolism of xenobiotics by cytochrome P450
p53 signaling pathway
Longevity regulating pathway − multiple species
Legionellosis
Ovarian steroidogenesis
Malaria
African trypanosomiasis
Nitrogen metabolism
Pathway name
Rich factor
0.01 0.02 0.03
Gene number
100
200
300
q value
5
4
3
2
1
C
DNP 4w VS. non-DNP
DNP 8w VS. non-DNP
DNP 8w VS. DNP 4w
6
3
14
0
11
3
6
D
DNP 4w VS. Vehicle
DNP 8w VS. Vehicle
DNP 4w VS. non-DNP
DNP 8w VS. non-DNP
10
8
3
0
1
3
2
0
14
7
2
0
0
3
0
